# Prevalence, Predictors, and Successful Treatment Outcomes of Xpert MTB/RIF–identified Rifampicin-resistant Tuberculosis in Post-conflict Eastern Democratic Republic of the Congo, 2012–2017: A Retrospective Province-Wide Cohort Study

**DOI:** 10.1093/cid/ciy1105

**Published:** 2019-02-13

**Authors:** André N H Bulabula, Jenna A Nelson, Eric M Musafiri, Rhoderick Machekano, Nadia A Sam-Agudu, Andreas H Diacon, Maunank Shah, Jacob Creswell, Grant Theron, Robin M Warren, Karen R Jacobson, Jean-Paul Chirambiza, Dieudonné Kalumuna, Bertin C Bisimwa, Patrick D M C Katoto, Michel K Kaswa, Freddy M Birembano, Liliane Kitete, Martin P Grobusch, Zacharie M Kashongwe, Jean B Nachega

**Affiliations:** 1 Department of Global Health, Division of Health Systems and Public Health, Unit for Infection Prevention and Control, Faculty of Medicine and Health Sciences, Stellenbosch University; 2a Infection Control Africa Network, Cape Town, South Africa; 2 Department of Epidemiology, University of Pittsburgh Graduate School of Public Health, Pennsylvania; 3 National Tuberculosis Program, Provincial Leprosy and Tuberculosis Coordination, South Kivu Branch, Bukavu, Democratic Republic of the Congo; 4 Department of Global Health, Center for Evidence-Based Health Care, Biostatistics Unit, Faculty of Medicine and Health Sciences, Cape Town, South Africa; 5 International Research Center of Excellence and Pediatric and Adolescent Human Immunodeficiency Virus Unit, Institute of Human Virology Nigeria, Abuja; 6 Division of Epidemiology and Prevention, Institute of Human Virology, University of Maryland School of Medicine, Baltimore; 7 Division of Medical Physiology, Faculty of Medicine and Health Sciences, Stellenbosch University, Cape Town, South Africa; 8 Center for Tuberculosis Research & Division of Infectious Diseases, Johns Hopkins School of Medicine, Baltimore, Maryland; 9 Stop TB Partnership, TB REACH Initiative, Geneva, Switzerland; 10 South African Department of Science and Technology and the National Research Foundation, Centre of Excellence for Biomedical Tuberculosis Research and South African Medical Research Council Centre for Tuberculosis Research, Division of Molecular Biology and Human Genetics, Faculty of Medicine and Health Sciences, Stellenbosch University, Cape Town, South Africa; 11 Department of Medicine, Division of Infectious Diseases, Boston University School of Medicine, Massachusetts; 12 Biomedical Laboratory Professor A. Z. Lurhuma, Mycobacterium Unit, Université Catholique de Bukavu, Democratic Republic of the Congo; 13 Centre for Environment and Health, Department of Public Health and Primary Care, Laboratory of Pulmonology, The Katholieke Universiteit Leuven, Belgium; 14 Department of Internal Medicine, Faculty of Medicine, Catholic University of Bukavu; 15 The Union Against Tuberculosis and Lung Diseases, Challenge Tuberculosis Initiative, Bukavu, Democratic Republic of the Congo; 16 Center of Tropical Medicine and Travel Medicine, Department of Infectious Diseases, Amsterdam University Medical Centers, The Academic Medical Center, The Netherlands; 17 Department of Medicine, University Hospital of Kinshasa, Democratic Republic of the Congo; 18 Department of Medicine and Center for Infectious Diseases, Faculty of Medicine and Health Sciences, Stellenbosch University, Cape Town, South Africa; 19 Departments of Epidemiology and International Health, Johns Hopkins Bloomberg School of Public Health, Baltimore, Maryland; 20 International Centre for Advanced Research and Training, Panzi, Bukavu, Democratic Republic of the Congo

**Keywords:** multidrug-resistant TB, prevalence, predictors, treatment outcomes, eastern DR Congo

## Abstract

**Background:**

Multidrug-resistant tuberculosis (MDR-TB) jeopardizes global TB control. The prevalence and predictors of Rifampicin-resistant (RR) TB, a proxy for MDR-TB, and the treatment outcomes with standard and shortened regimens have not been assessed in post-conflict regions, such as the South Kivu province in the eastern Democratic Republic of the Congo (DRC). We aimed to fill this knowledge gap and to inform the DRC National TB Program.

**Methods:**

of adults and children evaluated for pulmonary TB by sputum smear microscopy and Xpert MTB/RIF (Xpert) from February 2012 to June 2017. Multivariable logistic regression, Kaplan–Meier estimates, and multivariable Cox regression were used to assess independent predictors of RR-TB and treatment failure/death.

**Results:**

Of 1535 patients Xpert-positive for TB, 11% had RR-TB. Independent predictors of RR-TB were a positive sputum smear (adjusted odds ratio [aOR] 2.42, 95% confidence interval [CI] 1.63–3.59), retreatment of TB (aOR 4.92, 95% CI 2.31–10.45), and one or more prior TB episodes (aOR 1.77 per episode, 95% CI 1.01–3.10). Over 45% of RR-TB patients had no prior TB history or treatment. The median time from Xpert diagnosis to RR-TB treatment initiation was 12 days (interquartile range 3–60.2). Cures were achieved in 30/36 (83%) and 84/114 (74%) of patients on 9- vs 20/24-month MDR-TB regimens, respectively (*P* = .06). Predictors of treatment failure/death were the absence of directly observed therapy (DOT; adjusted hazard ratio [aHR] 2.77, 95% CI 1.2–6.66) and any serious adverse drug event (aHR 4.28, 95% CI 1.88–9.71).

**Conclusions:**

Favorable RR-TB cure rates are achievable in this post-conflict setting with a high RR-TB prevalence. An expanded Xpert scale-up; the prompt initiation of shorter, safer, highly effective MDR-TB regimens; and treatment adherence support are critically needed to optimize outcomes.

## Teaser

In tuberculosis (TB) patients from post-conflict eastern Democratic Republic of the Congo, 11.1% had rifampicin-resistant TB, of whom 46.5% had no prior TB. Cures were achieved in 83% and 74% of patients on 9- and 20/24-month multidrug-resistant TB regimens, respectively.

Multidrug-resistant tuberculosis (MDR-TB), defined as resistance to rifampicin and isoniazid, poses a global threat to TB control efforts [1, 2]. In contrast to downward trends in 30 high-burden countries, the Democratic Republic of the Congo (DRC) has had persistently high TB incidences and mortality rates [2]. In 2017, among the 81 million people in DRC, there was an estimated TB incidence of 322/100 000 and an estimated 60 000 TB-related deaths [2]. Estimated DRC MDR-TB rates were 2.2% among new cases and 9.7% in retreatment cases [2]. Limited laboratory infrastructure for both MTB cultures and drug susceptibility testing (DST) makes the determination of the actual MDR-TB burden and of treatment outcomes challenging [3, 4].

The second Congo war, in the aftermath of the 1994 Rwandan genocide, caused a massive disruption of health-care services in the eastern DRC [5]. In 2012, South Kivu was the first province of eastern DRC to roll out the Xpert MTB/RIF assay (Xpert; Cepheid, Sunnyvale, CA), a rapid genotypic assay for rifampicin susceptibility via *rpoB* mutation detection [6–8]. Laboratory capacities for Xpert were established at 10 TB diagnostic and treatment centres (CSDTs) in South Kivu province through the Stop TB Partnership’s TB REACH initiative [9]. Creswell et al reported a 5.3% prevalence of Xpert-diagnosed rifampicin-resistant TB (RR-TB) in South Kivu [10], compared to a 9.6% prevalence among smear-negative TB cases in the capital city Kinshasa, as reported by Mbonze et al [11]. Both studies focused on strategies to improve finding TB cases and on early Xpert user experiences, but did not document the risk factors for, or clinical outcomes of, RR-TB treatment.

We aimed to assess the prevalence and predictors of RR-TB, as well as treatment outcomes, to inform the strategic plan of the DRC National TB Program (NTP) and guide models development to improve MDR-TB care in low- and middle-income countries (LMICs).

## METHODS

### Study Design, Setting, and Population

**Figure 1.**
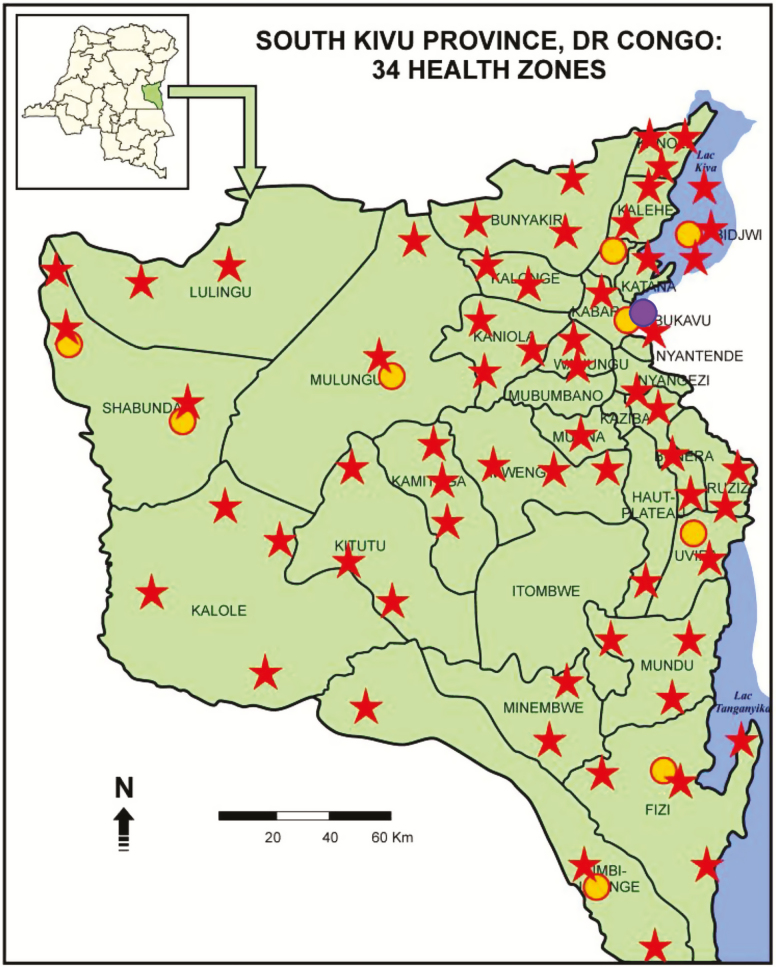
Tuberculosis diagnostic and treatment centers (red stars), plus those equipped with Xpert MTB/RIF (yellow dots) as well as GenoType MTBDR*sl* (purple dot) machines within the 34 health zones of the South Kivu Province, Democratic Republic (DR) of the Congo.

We performed a secondary analysis of data collected routinely from adults and children and evaluated for suspected pulmonary TB at 10 Xpert-equipped CSDTs serving 34 health zones of the South Kivu province from 1 February 2012 to 30 June 2017 (Figure 1). While described as post-conflict, the eastern DRC is still considered unstable, given the continued presence of multiple active militias [5]. TB cases were found and identified either passively (by referral) or actively (via symptom screening by community health workers [CHWs]). Ziehl-Neelsen sputum smear microscopy for acid-fast bacilli (AFB) and Xpert were performed on different sputum samples. Human immunodeficiency virus (HIV) status was not documented systematically, due to out-of-stock test kits.

### Treatment Regimens and Monitoring

Previously untreated, rifampicin-susceptible TB (RS-TB) patients received the standard, 6-month, first-line drug regimen, including 2 months of rifampicin (*R*), isoniazid (*H*), pyrazinamide (*Z*), and ethambutol (*E*), followed by 4 months of *R* and *H* (*2RHZE/4RH*). Retreatment cases after failure, relapse, or loss to follow-up (LTFU) received an 8-month regimen, including streptomycin (*S*) for the first 2 months (*2SRHZE/1RHZE/5RHE*) [11]. As recommended by World Health Organization (WHO) for reporting purposes, we considered RR-TB as a proxy for MDR-TB, because rifampicin mono-resistant TB has been considered relatively rare [2]. MDR-TB regimens included the standard, 9-month, shorter regimen with moxifloxacin (*Mfx*), clofazimine (*Cfz*), *E,* and *Z* throughout, supplemented by kanamycin (*Km*), prothionamide (*Pto*), and high-dose isoniazid (*H*_*h*_) during a 4-month intensive phase (*4KmMfxPtoCfzZH*_*h*_*E/5MfxCfzZE*) [12]. This regimen became available to our study cohort in 2016. A GenoType MTBDR*sl* (Hain LifeScience GmbH, Nehren, Germany) assay [11] was performed at the Biomedical Laboratory Professor A. Z. Lurhuma, Université Catholique de Bukavu, Bukavu (Figure 1), to exclude resistance to *Mfx* or *Km*, as per 2016 WHO guidelines on use of the shorter MDR-TB regimen [13]. The individualized (conventional) 20- or 24-month MDR-TB regimen included ofloxacin (*Ofx*) or levofloxacin (*Lfx*) and cycloserine (*Cs*) throughout (*6KmOfxPtoCsEZ/18OfxPtoCsEZ* or *6KmLfxPtoCsEZ/14LfxPtoCsEZ*, respectively) and was for RR-TB patients recruited before 2016 or not eligible for the 9-month regimen.

Patient responses to MDR-TB treatment were monitored monthly during the intensive phase and every 2 months during the continuation phase, with a sputum smear for AFB (if positive at baseline) and/or mycobacterial culture most frequently done on traditional Löwenstein–Jensen solid media. The BACTEC Mycobacteria Growth Indicator Tube liquid cultures (Becton Dickinson, Sparks, MD) were performed only occasionally, due to their relatively high cost. Monitoring for drug toxicity included baseline and follow-up clinical assessments; electrolyte, liver (if symptomatic), renal, and thyroid function tests; and visual acuity and audiometric assessments, per DRC NTP guidelines [12, 14]. MDR-TB patients were not hospitalized unless medically indicated. Directly observed therapy (DOT) was conducted by a nurse at the nearest CSDT, either daily during the intensive phase or weekly during the continuation phase; on the remaining days of the week, home-based DOT was performed by a trained CHW or an assigned family member. Of note, patients were provided incentives—food parcels, for nutritional support, and transportation subsidies (US $30/per month)—to facilitate their daily or weekly attendance at CSDTs for DOT [12].

### Treatment Outcomes Definitions

WHO definitions were used for MDR-TB treatment outcomes [13]. Cured indicates a patient who has completed treatment and has at least 3 consecutive, negative sputum smears for AFB or MTB cultures, with at least 30 days in between. Treatment completion indicates a patient who has completed treatment, but does not have bacteriologic confirmation. Death indicates a patient who dies for any reason during the course of treatment. Failure indicates a patient with (1) a positive sputum smear for AFB or MTB culture at 5 months or later after initiation of MDR-TB regimen, or (2) a bacteriological reversion to positive after conversion. Treatment success includes patients with cures or treatment completion, while unsuccessful treatment includes patients who died, failed treatment, or relapsed. LTFU refers to a patient lost to follow-up for 2 or more consecutive months. Of note, the WHO benchmarks for treatment successes and cures among TB patients, regardless of their drug resistance statuses, are ≥90% and >80%, respectively [2].

### Statistical Analysis

Data were summarized using proportions and medians (interquartile range [IQR]) for categorical and continuous variables, respectively. Chi-square tests and Wilcoxon rank sum tests were applied for tests of association, where appropriate. Using logistic regression models, we estimated the unadjusted association between individual, baseline characteristics and RR-TB. We estimated adjusted associations by including all baseline covariates *a priori* in a multivariate model. Starting with a full model, we then used a backward elimination procedure, excluding predictor variables with a *P* value < .1, and compared the estimated reduced model adjusted odds ratios (aOR) and associated 95% confidence intervals (CIs) to the full multivariate model estimates. The final model was based on variables with statistical significance in both the full and reduced models, plus variables known to have clinical significance. Patients who were LTFU were censored at the date of last appointment. Treatment failure was summarized using Kaplan–Meier estimates, and TB treatment survival was compared between regimens using log-rank tests. Univariable and multivariable Cox regression modelling identified those predictors independently associated with the hazard of treatment success or failure. Adjusted hazard ratios (aHR) and their associated 95% CIs were calculated to summarize the strength of associations between patient characteristics and treatment failure. For each baseline characteristic, we evaluated the proportional hazards assumption by checking for parallel lines in plots of –log{-log(survival)} versus log(time) for each category. For the final multivariate models, we performed a universal test of proportional hazards based on Schoenfeld residuals. All *P* values that we report are exact and two-tailed; a value less than .05 was considered statistically signiﬁcant. Stata software version 13.1 (Stata, College Station, TX) was used for all statistical analyses.

### Ethics Approvals

This study was approved by the Health Research Ethics Committees of Stellenbosch University, Cape Town, South Africa (reference number S15/03/059) and the Université Catholique de Bukavu’s Referral Provincial Hospital, Bukavu, South Kivu Province, DRC (reference number 25.042/946/Staff/LK/HPGRB/BKV/014).

## RESULTS

### Baseline Demographics and Clinical Characteristics

**Figure 2.**
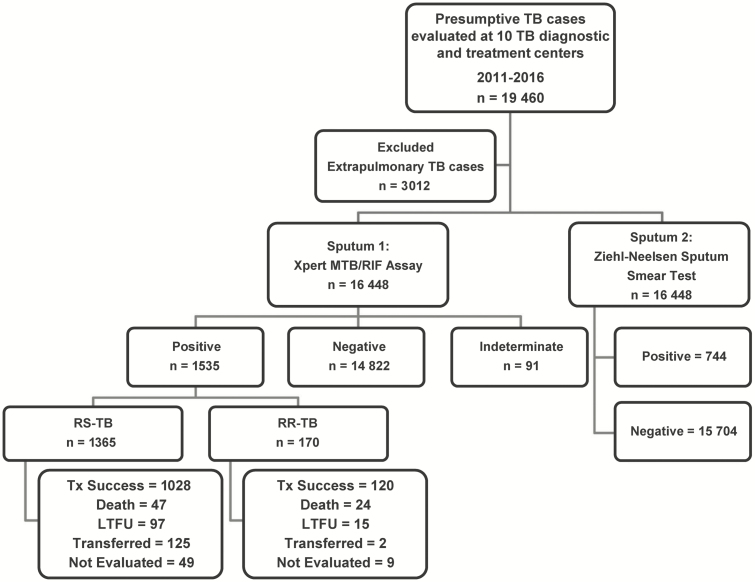
Study flow diagram. Abbreviations: LTFU, loss to follow-up; RR, rifampicin resistant; RS, rifampicin susceptible; TB, tuberculosis; Tx, treatment.

Figure 2 displays the patient flow for all 10 CSDTs during the study period: 16 488 patients with presumptive pulmonary TB were screened by Xpert, capturing over 90% of those diagnosed with TB during the study period from these CSDTs. Their age range was 1–98 years, their median age was 35 years (IQR 24–52), and 55.2% were male. Ultimately, 1535 (9.3%) patients were confirmed by Xpert or culture to have TB. The median time from a positive Xpert result to TB treatment initiation was significantly longer for RR-TB vs RS-TB patients (12.0 [IQR 3.0–60.2] days vs. 1.0 [IQR 0.0–4.4] days, respectively; *P* < .001; Table 1).

**Table 1.** Baseline Demographic and Clinical Characteristics by Xpert MTB/RIF Assay Result

	Overall	TB Negative	TB Positive	RS-TB	RR-TB
Sample size, n (%)	16 357 (100)^a^	14 822 (90.6)	1535 (9.4)	1365 (88.9)^b^	170 (11.0)^b^
Median age, years (IQR)	36 (24–52)	37 (24–52)	35 (26–45)	35 (26–45)	35 (27–44)
Age <20 years	2648 (16.2%)	2481(16.7%)	167 (10.9%)	153 (11.2%)	14 (8.2%)
Age >20 years	13 709 (83.8%)	12 341 (83.3%)	1368 (89.1%)	1212 (88.8%)	156 (91.8%)
Male sex, n (%)	9029 (55.2)	8028 (54.2)	1001 (65.2)	886 (64.9)	115 (67.6)
HIV status, n (%)					
Negative	994 (6.1)	29 (0.2)	965 (62.8)	843 (61.7)	122 (71.7)
Positive	135 (0.8)	28 (0.2)	107 (6.9)	91 (6.7)	16 (9.4)
Unknown	15 228 (93.1)	14 765 (99.6)	463 (30.1)	431 (31.6)	32 (18.8)
WHO HIV stage, n (%)					
Stage I-III	95 (67.4)	7 (25.0)	88 (82.2)	76 (83.5)	12 (75.0)
Stage IV	12 (8.9)	0 (0.0)	12 (11.2)	8 (8.8)	4 (25.0)
Missing data	28 (20.7)	21 (75.0)	7 (6.5)	7 (7.7)	0 (0.0)
TB category, n (%)					
New cases	…	…	1289 (84.0)	1217 (90.0)	72 (42.4)
Retreatment	…	…	234 (15.2)	136 (10.0)	98 (57.6)
After failure	…	…	66 (4.2)	35 (2.6)	31 (18.2)
After relapse	…	…	105 (6.8)	65 (4.8)	40 (23.5)
After LTFU	…	…	63 (4.1)	36 (2.7)	27 (15.9)
Missing data	…	…	12 (0.8)	12 (0.9)	0 (0.0)
Prior TB Episode, n (%)					
0	…	…	1296 (84.4)	1217 (89.9)	79 (46.5)
1	…	…	185 (12.0)	121 (8.9)	64 (37.6)
≥2	…	…	42 (2.7)	15 (1.1)	27 (15.8)
Missing data	…	…	12 (0.8)	12 (0.9)	0 (0.0)
Median (IQR) days from Xpert MTB/RIF result to treatment	…	…	1.6 (0–6.2)	1.1 (0–4.4)	12 (3–62.4)
Positive sputum smear microscopy for AFB, n (%)	715 (4.5)	92 (0.6)^c^	623 (40.5)	499 (36.6)	124 (72.9)

Abbreviations: AFB, acid-fast bacilli; HIV, human immunodeficiency virus; IQR, interquartile range; LTFU, lost to follow-up; MDR, multidrug resistant; RR, rifampicin resistant; RS, rifampicin susceptible; S, success; TB, tuberculosis; WHO, World Health Organization.

^a^This total excludes the indeterminate Xpert MTB/RIF assay results.

^b^This percentage uses TB-positive cases (n = 1535) as a denominator.

^c^Possible contamination by non-tuberculous Mycobacteria.

### Prevalence and Predictors of Rifampicin Resistance Tuberculosis

Of the 1535 Xpert MTB-positive patients, 170 (11.1%, 95% CI 9.5–12.8) had RR-TB; 46.5% of those with RR-TB had no prior TB episode. The prevalence of RR-TB was lowest among new TB cases (5.6%), but for previous treatment failure, relapse, or LTFU cases, the prevalences of RR-TB were 47%, 38.1%, and 42.9%, respectively (Table 1). RR-TB was significantly more likely among retreatment vs new cases (aOR 4.92, 95% CI 2.31–10.45) and positive sputum smear cases (aOR 2.23, 95% CI 1.49–3.32). For each prior TB episode, the risk of RR-TB was increased nearly 2-fold. Age, sex, and HIV status were not associated with RR-TB (Table 2).

**Table 2.** Univariable and Multiple Logistic Regression Analysis of Predictors for Rifampicin-resistant Tuberculosis

Variable	Total, N	RR-TB cases, n (%)	Crude Odds Ratio (95% CI)	*P* Value	Adjusted Odds Ratio (95% CI)	*P* Value
**Overall**	1535	170 (11.0)	…	…	…	…
**Age, years**						
<20	167	14 (8.4)	1.00	…	…	…
20–24	168	19 (11.3)	1.39 (.67–2.88)	.370	…	…
25–39	635	80 (12.6)	1.58 (.87–2.86)	.868	…	…
40–59	438	47 (10.7)	1.31 (.70–2.46)	.393	…	…
>60+	127	10 (7.9)	0.93 (.40–2.18)	.875	…	…
**Sex**						
Female	534	55 (10.3)	1.00	…	…	…
Male	1001	115 (11.5)	1.13 (.80–1.59)	.480	…	…
**HIV status**						
Negative	965	122 (12.6)	1.00	…	1.00	…
Positive	107	16 (15.0)	1.21 (.69–2.13)	.499	0.90 (.47–1.72)	.756
Unknown	463	32 (6.9)	0.49 (.32–0.74)	.001	0.59 (.37–.93)	.024
**Sputum AFB**						
Negative	912	46 (5.0)	1.00	…	1.00	…
Positive	623	124 (19.9)	4.68 (3.28–6.68)	<.001	2.23 (1.49–3.32)	<.001
**TB Category**						
New cases	1289	72 (5.6)	1.00	…	1.00	…
Retreatment cases	234	98 (41.9)	4.68 (3.28–6.68)	<.001	4.92 (2.31–10.45)	<.001
**Prior TB**						
Per 1 prior Episode	…	…	6.34 (4.77–8.44)	<.001	1.74 (.98–3.10)	<.060

Abbreviations: AFB, acid-fast bacilli; CI, confidence interval; HIV, human immunodeficiency virus; RR, rifampicin resistant; TB, tuberculosis.

### Treatment Outcomes

**Figure 3.**
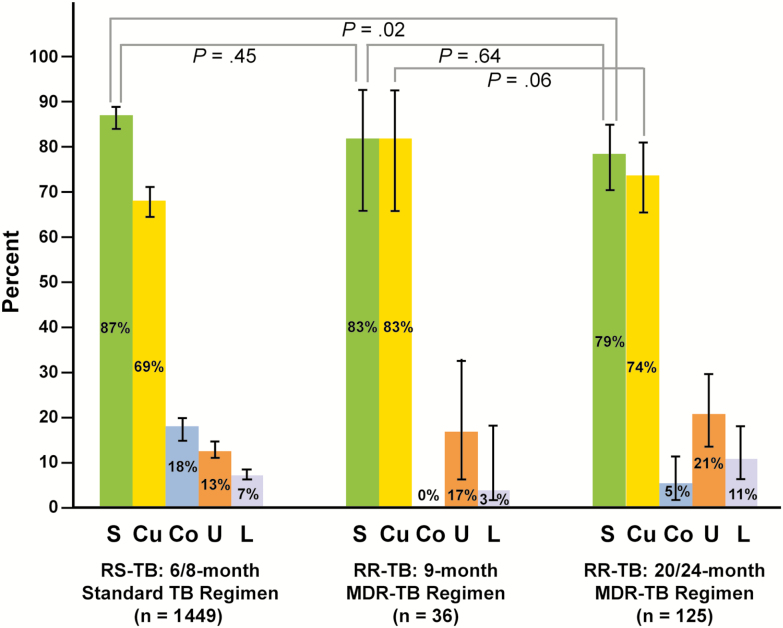
Treatment outcomes, comparing patients with RS-TB treated with a 6/8-month standard TB treatment regimen to patients with RR-TB treated with a 9- or 20/24-month MDR-TB regimen. Abbreviations: Co, treatment completion without bacteriologic confirmation; Cu, cured or treatment completion with bacteriologic confirmation; L, lost to follow-up; MDR, multidrug resistant; RR, rifampicin resistant; RS, rifampicin susceptible; S, success; TB, tuberculosis; U, unsuccessful (failure or death).

TB treatment was initiated in 1503 of 1535 (98%) patients. When 6- or 8-month standard regimens were administered, 1028 of 1184 (87%) RS-TB patients were treated successfully. Of the 170 patients with RR-TB, 68 (40%), 57 (34%), and 36 (21%) were treated with the 24-, 20-, and 9-month MDR-TB regimens, respectively; 9 (5%) were LTFU or dead before treatment initiation. The success rate of the 9-month regimen was 30/36 (83%). The 20/24-month regimen achieved success and cures in 90/114 (79%) and 84/114 (74%) of evaluable patients, respectively (Figure 3). Treatment successes (*P* = .64) or cures (*P* = .06) following treatment for RR-TB did not differ significantly between the 9- or 20/24-month regimens. Of note, there was no *mfx* and *km* resistance documented (0/36, 0.0%) for RR-TB patients screened by GenoType MTBDR*sl* assay for the shorter MDR-TB regimens.

**Figure 4.**
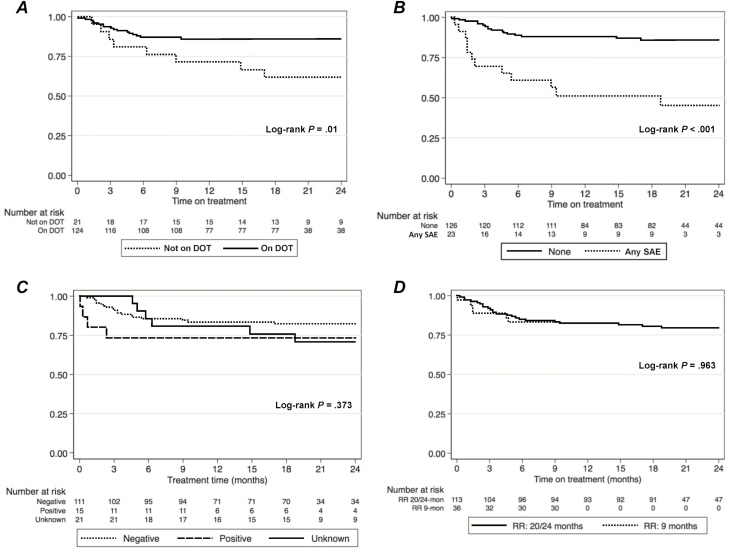
Kaplan–Meier plots showing time to death or failure, stratified by: (*A*) DOT status; (*B*) grade III-IV SAEs; (*C*) HIV status (negative vs positive vs unknown); (*D*) by 9- vs 20/24-month MDR-TB regimens. Abbreviations: DOT, directly observed therapy; HIV, human immunodeficiency virus; MDR, multidrug resistant; RR, rifampicin resistant; SAE, serious adverse event; TB, tuberculosis.

Serious adverse events (SAEs; grade III-IV) were more common among RR- vs RS-TB patients (16.2% vs 0.7%, respectively; *P* < .001). Among 159 RR-TB patients, SAEs were reported as follows: ototoxicity (n = 13, 8%), renal failure (n = 4, 2.4%), hepatitis (n = 4, 2.4%), anaemia (n = 2, 1.2%), and optic neuritis (n = 1, 0.5%). Having any SAE was independently associated with a more than 4-fold increase in the hazard of treatment failure (Table 3). There was a significant difference in the time to either death or treatment failure, relative to the initiation of treatment by DOT (aHR 3.42, 95% CI 1.34–8.72) and any SAE (aHR 4.81, 95% CI 2.04–11.30; Figures 4A and B), but not when comparing by HIV status (aHR 2.83, 95% CI 0.77–10.39; Figure 4C) or by 9- vs 20/24-month MDR-TB regimens (aHR 1.02, 95% CI 0.41–2.56; Figure 4D).

**Table 3.** Univariable and Multiple Cox Regression Analysis of Predictors Associated With Treatment Failure or Death Among Rifampicin-resistant Tuberculosis Patients

Variable	N	Died or Failed, n (%)	Crude HR (95% CI)	*P* Value	Adjusted HR (95% CI)	*P* Value
Age, years						
<20	13	3 (23.1)	1.00	…	…	…
20–24	18	6 (33.3)	1.67 (.31–9.14)	.552	…	…
25–39	74	18 (24.3)	1.41 (.32–6.17)	.647	…	…
40–59	44	9 (20.4)	.92 (.18–4.54)	.914	…	…
>60+	10	3 (30.0)	1.54 (.22–10.92)	.667	…	…
Sex						
Female	50	17 (34.0)	1.00	…	…	…
Male	109	22 (20.1)	.53 (.26–1.11)	.091	…	…
HIV Status						
Negative	117	25 (21.4)	1.00	…	1.00	…
Positive	16	5 (31.2)	1.191 (.65–5.62)	.239	2.83 (.77–10.39)	.116
Unknown	24	9 (37.5)	1.56 (.62–3.92)	.340	0.74 (.23–2.40)	.622
Sputum smear microscopy for AFB						
Negative	39	11 (28.2)	1.00	…	…	…
Positive	120	28 (23.3)	.87 (.38–1.97)	.748	…	…
TB category						
New cases	64	18 (28.1)	1.00	…	…	…
Re-treatment cases	95	21 (22.1)	.58 (.28–1.22)	.152	…	…
TB episode number						
Per 1 prior episode of TB	159		.67 (.39 - 1.15)	.143	…	…
Days from result to treatment	141		1.00 (.99–1.00)	.687	…	…
MDR-TB regimen						
20/24-month	114	24 (21.1)	1.00	…	…	…
9-month	36	6 (16.7)	1.02 (.41–2.56)	.963	…	…
DOT						
No	26	13 (50.0)	1.00	…	1.00	…
Yes	124	17 (13.7)	.35 (.15–.81)	<.001	.29 (.11–.74)	.010
SAEs						
None	134	25 (18.7)	1.00	…	…	…
Any SAE	25	14 (56.0)	5.08 (2.42–10.66)	<.001	4.81 (2.04–11.30)	<.001

Abbreviations: AFB, acid-fast bacilli; CI, confidence interval; DOT, directly observed therapy; HIV, human immunodeficiency virus; HR, hazard ratio; MDR, multidrug resistant; SAE, serious adverse event; TB, tuberculosis.

## DISCUSSION

We observed a high prevalence (11.1%) of RR-TB among nearly 1600 adults and children with Xpert-positive MTB in the South Kivu province. Independent predictors of RR-TB were sputum smear positivity and retreatment TB, and 46.5% of RR-TB patients had no prior TB episode. High proportions of RR-TB patients on 9- (83%) and 20/24- month (74%) MDR-TB regimens were cured (*P* = .06). Independent predictors of treatment failure/death while on an RR-TB regimen were the lack of DOT and the occurrence of any SAE.

Our data are consistent with those of Mbonze et al, who found a high prevalence (9.6%) of RR-TB among 991 samples from Kinshasa [11]. Creswell et al, however, in their TB REACH report analyzing data from three South Kivu sites, found that only 5.3% (n = 567) of samples that tested positive by Xpert were RR-TB [10]. Factors accounting for the differences between our study and that of Creswell include our larger sample size and coverage area, the longer observation time, and the greater number of retreatment cases tested.

Patients with RR-TB were more likely to have positive sputum smears for AFB and to be retreatment TB cases. The latter situation could be a consequence of the prior lack of DST, due to limited laboratory capacities and leading to empiric RS-TB treatment. Inadequate treatment leads to progressive TB and the presence of persistently sputum smear–positive individuals in the community, with increased TB transmission as strains have more opportunities to mitigate fitness costs through compensatory mutations [15, 16]. We also found that even one prior TB treatment episode was associated with a roughly 80% increase in the odds of acquiring RR-TB, which is in agreement with several other reports from LMICs [17–19].

Importantly, we documented the evidence for transmitted RR-TB, since over 40% of those with RR-TB had no prior TB episode (Table 1). This is in line with published empiric and modelling data suggesting that, in high TB burden settings, many cases of RR-TB result from the transmission of drug-resistant strains of TB, rather than from acquired resistance [2, 20, 21]. Therefore, improving the diagnosis, prevention, and treatment strategies of both RS- and RR-TB cases, including access to genotypic DST and shorter regimens, is critical [22].

Of concern, the median time from a positive Xpert result to treatment initiation was delayed to nearly 60 days for more than 25% of patients with RR-TB, due to out-of-stock drugs and supply chain issues of second-line drugs (SLDs), as they need to be transported from the capital city of Kinshasa to the remote CSDTs. To maximally benefit from Xpert testing, these important logistical and structural challenges must be addressed by the DRC’s NTP and stakeholders.

We did not find any significant association between HIV infection and RR-TB prevalence, treatment failure, or mortality; however, our study had limited statistical power for such associations, most likely due to the low HIV prevalence (<5%) in the South Kivu province and/or the 18.8% of RR-TB patients not tested for HIV. While there are conflicting reports about associations between HIV and MDR-TB prevalence [23–25], the negative impact of HIV on MDR mortality is well established [26]. Indeed, in a meta-analysis including 2725 HIV-infected adults and children with MDR-TB, Isaakidis et al found that only 57% were treated successfully, and the mortality among HIV-infected adults was 4 times higher than that of HIV-negative populations (38% vs 11%), while mortality among children was twice as high (11.5% vs 6%) [26].

We documented similar programmatic treatment success with 9- and 20/24-month MDR-TB regimens. These results are in line with those reported from observational studies in Bangladesh and sub-Saharan Africa [27–33], as well as the interim results of the standardised treatment regimen of anti-TB drugs for patients with MDR-TB (STREAM) Stage 1 trial, which showed that both the shorter and longer MDR-TB regimens achieved success in roughly 80% of participants [34, 35]. The documented 0.0% MTB resistance to *Mfx* and *Km* in our setting may account, in part, for this favorable outcome. Also, our data, similar to those of others, show that SAEs are associated with unsuccessful MDR-TB outcomes [36]. Indeed, prevalent SAEs are associated with the toxicity of second-line injectable drugs: that is, renal failure or ototoxicity associated with *Km*. In the STREAM Stage 2 trial, second-line injectable drugs were replaced by a fully oral, bedaquiline (BDQ)-containing regimen, one of the two new (along with delamanid [DLM]) potent MDR-TB drugs [37, 38] that are now being prescribed increasingly in LMICs following compassionate pilot access programs [39]. In August 2018, the WHO recommended that initiation of the shorter regimen be conditional to: (1) availability of DST, to exclude resistance to at least *Mfx* and *Km*, and (2) close monitoring of the patient’s safety, as well as their treatment response [13, 35]. Switching non-responding patients (or those experiencing SAEs) to alternative SLDs and/or new regimens needs to be based on the new regrouping of MDR-TB drugs, ideally all oral, including BDQ or DLM [35].

Finally, we found that treatment under DOT was significantly associated with a decreased hazard of death/failure among RR-TB patients, as also reported in the meta-analysis by Ahuja et al [40]. However, it is likely that DOT is facilitated by socioeconomic incentives that, in turn, minimize LTFU and optimize patient outcomes, as also reported by Charles et al in post-earthquake Port-au-Prince, Haiti [41]. Of note, a meta-analysis by Richterman et al showed that patients receiving cash incentives during treatment for active RS-TB experienced improved clinical outcomes [42], but an assessment of the efficacy and cost-effectiveness of socioeconomic incentives for MDR-TB patients is needed.

Our data have important clinical/public health implications and can inform models of MDR-TB care in LMICs, including: (1) the scale-up of Xpert and genotype DST to identify earlier cases of MDR-TB among new and retreatment cases; (2) investments in socioeconomic incentives and training CHWs to deliver home-based DOT; (3) the integration of TB/HIV testing and treatment services; and (4) the need for household-based MDR-TB prevention strategies. However, confirmation of the impact of some of the above interventions will require prospective, interventional studies and cost-effectiveness analyses.

Our study has several strengths: (1) a detailed analysis of a large sample of Xpert-diagnosed TB cases in adults and children recruited from multiple TB diagnostic and treatment centers across the South Kivu province; (2) cohort data from a post-conflict setting, with multiple barriers to health care providing both feasibility data and MDR-TB treatment outcomes data relevant to TB clinicians and NTP implementers, as well as stakeholders in LMICs; (3) the identification of those NTP challenges requiring immediate attention (eg, timely SLD procurement; TB/HIV testing/treatment services integration); and (4) suggestions of areas for future intervention research and/or implementation science.

Our study also has several limitations: (1) the retrospective, cohort study design is subject to unmeasured confounding factors that were not adjusted for and to the possibility of recall bias or data capture errors, leading to the misclassification of information on prior TB episodes/treatment; (2) extended genotypic DST of other key first-line drugs or SLDs was not routinely affordable/available in this setting; (3) a limited number of RR-TB patients were treated with the 9-month MDR-TB treatment regimen; and (4) the unknown HIV status of 18.8% of RR-TB patients, due to the unavailability of HIV test kits. Despite these shortcomings, our study provides important, new, and significant findings about RR-TB epidemiology and treatment outcomes in a challenging setting.

## CONCLUSIONS

RR-TB was present in 11.1% of patients with confirmed TB in post-conflict DRC and was more frequent among patients with a positive sputum smear and retreatment TB. More than 45% of those with RR-TB had no prior TB episode, which highlights an urgent need for novel and effective household MDR-TB prevention strategies. Finally, we demonstrate that successful MDR-TB treatment is achievable in approximately 80% of patients in this setting. Scaling up the use of Xpert; the timelier initiation of shorter, safer, highly potent MDR-TB regimens; and consistent treatment adherence support are needed to optimize treatment outcomes.

## Notes

Presented in part: American Thoracic Society 2017 International Conference, Washington, DC, 19–24 May 2017. Poster #A2121.

Presented in part: 2nd International Research Symposium of the International Centre for Advanced Research and Training, Bukavu, Democratic Republic of the Congo, 17–19 August 2017. Oral Abstract #14.

***Author contributions.* **A. N. H. B. assisted with the protocol development, data extraction, data analysis, interpretation of results, and drafting of the manuscript. J. A. N., J. C., and A. H. D. assisted with the data analysis, writing of the manuscript, data interpretation, and revisions. J. B. N. assisted with the protocol development, statistical analyses, interpretation of results, writing of the manuscript, and revisions. R. M. helped with the data analysis, drafting of the manuscript, and revisions. Z. M. K. advised on the clinical and programmatic aspects of the manuscript. All authors reviewed the paper for additional interpretation, editing, and revisions and approved the final version.

***Acknowledgments.* **The authors thank John L. Johnson, Case Western Reserve University; Sarita N. Shah, US Centers for Disease Control and Prevention; Serena P. Koenig, Brigham and Women’s Hospital and Harvard Medical School; Kelly E. Dooley, Divisions of Clinical Pharmacology and Infectious Diseases, Johns Hopkins University; Gerald Friedland, Yale University; Helen Cox, University of Cape Town; Mark Nicol, Division of Medical Microbiology, University of Cape Town; Emmanuel André, Medical Microbiology, Université Catholique de Louvain, Brussels, Belgium; and Angela Dramowski, and H. Simon Schaaf, Stellenbosch University Faculty of Medicine and Health Sciences, Cape Town, South Africa for their critical review of the manuscript. We also express our sincere gratitude to Ms. Caroline E. Connor for editing services, and the laboratory staff of the National Tuberculosis Program, Provincial Leprosy and Tuberculosis Coordination, South Kivu Branch, Bukavu, Democratic Republic of the Congo, who performed Xpert MTB/RIF tests: Rosette L. Nyota, Tengeneza Itongwa, Andale Kale, Jean-Paul Muhanano, Sylvestre Ndariba, and Valery Badesire; as well as the dedicated field workers involved in data collection: Deo Muzigo and Aloise Fataki.

***Financial support.* **Xpert MTB/RIF testing infrastructure was funded by Global Affairs Canada through STOP TB Partnership, TB REACH Wave-2 grant to the South Kivu Branch of Democratic Republic of the Congo (DRC) National Tuberculosis Program in 2011 (coordinators: Dr. Emmanuel André and Dr. Dieudonné Kalumuna). The GenoType MTBDRsl assays were funded by Vlaamse Interuniversitaire Raad - Universitaire Ontwikkelingssamenwerking (VLIR-UOS) Institutional University Cooperation (IUC) supplemental grant number PRDC 2012MP80. This IUC grant supports a partnership between Ghent University, the University of Antwerp, Katholieke Universiteit Leuven in Belgium, and the Université Catholique de Bukavu in the DRC (coordinators: Professor Kanigula Mubgwa and Professor Wenceslas Ruhana Mirindi Busane).

***Potential conflicts of interest.* **J. B. N. is supported by research grants from the US National Institutes of Health (NIH)/National Institutes for Allergy and Infectious Disease, the AIDS Clinical Trial Group/Stellenbosch University Clinical Trial Unit (grant number 2UM1AI069521-08), and the Pittsburgh-Stellenbosch University AIDS-Comorbidities Training Research Program (NIH/Fogarty International Center; grant number 1D43TW010340-01). G.T. and R.M.W are partially funded by the South African government through the South African Medical Research Council. A. H. D. is supported by the National Research Foundation of South Africa. Z. M. K. and B. C. B. are supported by Universitaire Ontwikkelingssamenwerking (VLIR-UOS) (grant number PRDC 2012MP80). All other authors report no potential conflicts. All authors have submitted the ICMJE Form for Disclosure of Potential Conflicts of Interest. Conflicts that the editors consider relevant to the content of the manuscript have been disclosed.
